# Production of Minor Ginenosides from *Panax notoginseng* by Microwave Processing Method and Evaluation of Their Blood-Enriching and Hemostatic Activity

**DOI:** 10.3390/molecules23061243

**Published:** 2018-05-23

**Authors:** Huiying Liu, Jun Pan, Ye Yang, Xiuming Cui, Yuan Qu

**Affiliations:** 1 Faculty of Life Science and Technology, and Yunnan Provincial Key Laboratory of Panax Notoginseng, Kunming University of Science and Technology, Kunming 650500, China; lhying7836@163.com (H.L.); q270228@126.com (Y.Y.); sanqi37@vip.sina.com (X.C.); 2 Yunnan Provincal Academy of Agricultural Sciences, Kunming 650231, China; Pjun2017@126.com

**Keywords:** microwave processing, response surface methodology, minor ginsenosides, blood-enriching activity, hemostatic activity

## Abstract

A green solvent extraction technology involving a microwave processing method was used to increase the content of minor ginsenosides from *Panax notoginseng*. This article aims to investigate the optimization of preparation of the minor ginsenosides by this microwave processing method using single-factor experiments and response surface methodology (RSM), and discuss the blood-enriching activity and hemostatic activity of the extract of microwave processed *P. notoginseng* (EMPN) The RSM for production of the minor ginsenosides was based on a three-factor and three-level Box-Behnken design. When the optimum conditions of microwave power, temperature and time were 495.03 W, 150.68 °C and 20.32 min, respectively, results predicted that the yield of total minor ginsenosides (*Y*_9_) would be 93.13%. The actual value of *Y*_9_ was very similar to the predicted value. In addition, the pharmacological results of EMPN in vivo showed that EMPN had the effect of enriching blood in *N*-acetylphenylhydrazine (APH) and cyclophosphamide (CTX)-induced blood deficient mice because of the increasing content of white blood cells (WBCs) and hemoglobin (HGB) in blood. Hemostatic activity in vitro of EMPN showed that it had significantly shortened the clotting time in PT testing (*p <* 0.05). The hemostatic effect of EMPN was mainly caused by its components of Rh_4_, 20(*S*)-Rg_3_ and 20(*R*)-Rg_3_. This microwave processing method is simple and suitable to mass-produce the minor ginsenosides from *P. notoginseng*.

## 1. Introduction

The root of *Panax notoginseng* (Burk.) F. H. Chen is a well-known traditional Chinese medicine in China and around the world. Traditionally, raw notoginseng is commonly used in the treatment of trauma and blood circulation, while the steamed notoginseng can enrich the blood [1,2]. Both of them are not only different in treatment function, but also different in chemical composition. So far, more than 100 dammarane-type saponins have been isolated and identified from raw notoginseng, five major components of which are respectively ginsenosides Rg_1_, Re, Rb_1_, Rd and notoginsenoside R_1_ [3,4,5]. However, more than 50 new components are produced by dehydration, hydroxylation or epoxidation of the main saponins in the raw notoginseng after hydrolysis of the C-20 sugar moiety by the steaming process [6,7,8]. Eight minor ginsenosides of the steamed notoginseng—20(*S*)-Rh_1_, 20(*R*)-Rh_1_, Rh_4_, Rk_1_, Rk_3_, 20(*S*)-Rg_3_, 20(*R*)-Rg_3_ and Rg_5_—are less polar compounds [9,10,11]. These minor ginsenosides cause the functional differences between raw and steamed notoginseng.

So far, the minor ginsenosides have been difficult to obtain because of their low content, but some of them are good potential drug candidates based on their biological activity, such as the anti-tumor activity of ginsenoside compound K, ginsenoside Rg_3_ and ginsenoside Rk_1_ [12,13,14,15], anti-apoptotic activity of ginsenoside Rg_5_ [16], and antiviral activity of 20(*R*)-ginsenoside Rh_2_ [17]. Methods for obtaining minor saponins mainly include acid-base degradation [18], enzymatic degradation [19,20] and microbial transformation [21,22]. Some of the main ginsenosides can be transformed into minor ginsenosides by the steaming process. It is reported that the *P. notoginseng* steaming method is usually performed for a long time at a high temperature (48 h at 120 °C) [23]. However, there is a new method to increase the content of minor ginsenosides Rg_3_, Rg_5_, and Rk_1_ from the ginseng extract using a microwave processing method [24,25]. The microwave technique is widely used as a “green” solvent extraction technology [26,27]. Studies have showed that the microwave extraction technique had many advantages such as a faster extraction rate, reduced organic solvent consumption, and sample preparation at lower costs. Compared with the conventional steaming method, the microwave processing method is highly efficient process because of the shorter processing time. However, there are no reports in the production of minor saponins from *P. notoginseng* using the microwave processing method. The chemical components and pharmacological activities of transformed minor saponins from *P. notoginseng* with this processing method have not been studied.

In our study, the extracts of *P. notoginseng* containing minor ginsenosides were prepared by different microwave processing methods. As many factors affect the response, response surface methodology (RSM) was used to optimize the reaction conditions for production of the minor ginsenosides. The main effecting factors of microwave power, temperature and time were discussed in RSM based on single factor experiments. In addition, the blood-enriching activity and hemostatic activity of the extract of microwave processed *P. notoginseng* (EMPN) containing minor ginsenosides were analyzed in order to evaluate their potential medicinal value.

## 2. Results and Discussion

### 2.1. Structural Changes during the Microwave Processing of Saponins

**Figure 1:**
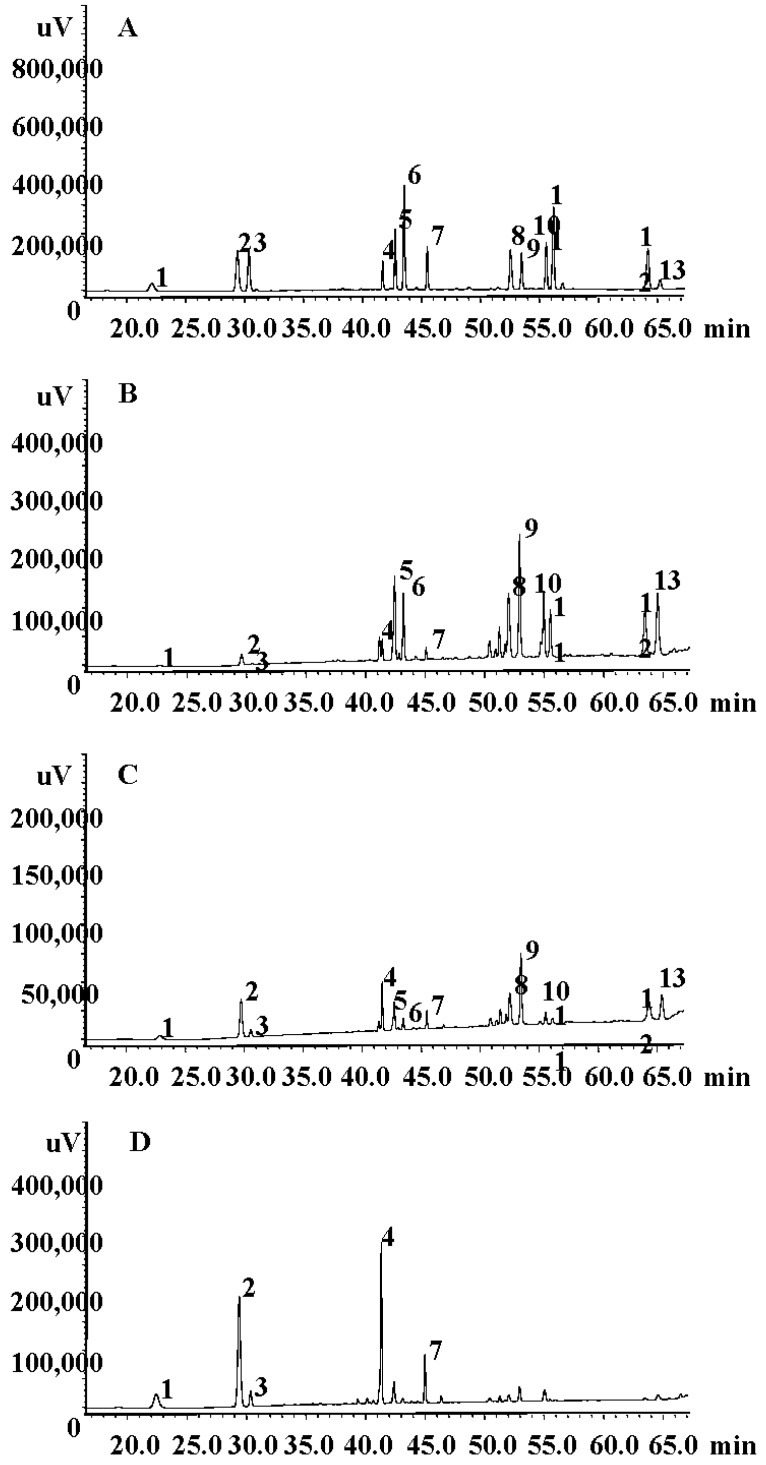
HPLC chromatograms of mixed standards (**A**); notoginseng processed by the microwave processing method at 500 W and 150 °C for 20 min (**B**); notoginseng steamed at 120 °C for 4 h (**C**) and raw notoginseng (**D**). Peaks: 1, notoginsenoside R_1_; 2, ginsenoside Rg_1_; 3, ginsenoside Re; 4, ginsenoside Rb_1_; 5, 20(*S*)-ginsenoside Rh_1_; 6, 20(*R*)-ginsenoside Rh_1_; 7, ginsenoside Rd; 8, ginsenoside Rk_3_; 9, ginsenoside Rh_4_; 10, 20(*S*)-ginsenoside Rg_3_; 11, 20(*R*)-ginsenoside Rg_3_; 12, ginsenoside Rk_1_; 13, ginsenoside Rg_5_.

The chemical ingredient changes of raw notoginseng and processed notoginseng are shown in the HPLC chromatograms in Figure 1. The main components of raw notoginseng are ginsenosides Rg_1_, Re, Rb_1_, Rd and notoginsenoside R_1_, which are obviously different from those of processed notoginseng. After steaming and microwave processing, the main constituents of raw notoginseng are decreased and transformed into eight minor ginsenosides, which were identified as 20(*S*)-Rh_1_, 20(*R*)-Rh_1_, Rh_4_, Rk_1_, Rk_3_, 20(*S*)-Rg_3_, 20(*R*)-Rg_3_ and Rg_5_ by comparing their retention times with standard ginsenosides [23]. When notoginseng was steamed at 120 °C for 4 h, most of saponins were transformed into minor ginsenosides except ginsenoside Rg_1_. Under the condition of microwave treatment of 500 W, 150 °C and 20 min, all the saponins were degraded into minor ginsenosides.

**Scheme 1:**
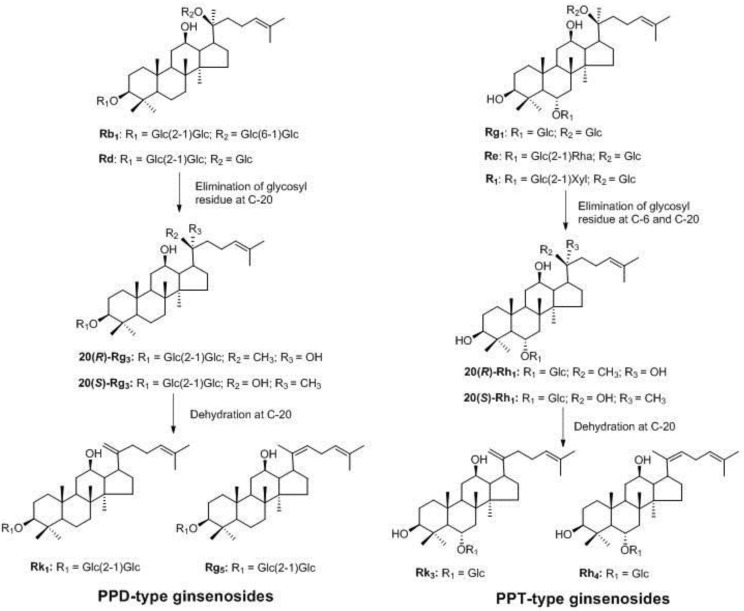
Proposed structural changes of saponins during the microwave processing of *P. notoginseng*. -Glc, d-glucopyranosyl; -Rha, l-rhamnopyranosyl; -Xyl, d-xylopyranosyl.

The principle and application of microwave dielectric heating in chemistry has been reported by Galema [28]. Microwave radiation induces molecular dipoles to orientate in the direction of electromagnetic field, and generates heat. The heating process occurs within the molecule, and is hence described as “inside heating”. If the microwave energy matches the rotation energy of polar molecules, the reactivity of polar bonds such as the glycosidic bond is increased and it becomes easy to break [29]. The structural changes of saponins in the microwave processing are shown in Scheme 1. When the microwave power and temperature increased, the glycosyl residues of major saponins were easily decreased. The protopanaxadiol (PPD) group, e.g., in ginsenosides Rb_1_ and Rd, was hydrolyzed at the glucosyl residue of C-20 to produce 20(*S*)/(*R*)-Rg_3_, which was then dehydrated at C-20 to yield Rk_1_ and Rg_5_. Similarly, the protopanaxatriol (PPT) group, such as found in ginsenosides Rg_1_, Re and notoginsenoside R_1_ easily lost the glycosyl residue at C-6 or C-20 to produce 20(*S*)/(*R*)-Rh_1_, and then Rh_1_ formed Rk_3_ and Rh_4_ through dehydration at C-20. During this process, it was evident that C-3 sugar moiety has higher temperature stability than the C-6 or C-20 sugar moieties. After elimination of the glycosyl residue at C-20, 20(*S*) and 20(*R*) epimers were produced by the selective attack of the OH group like 20(*S*)/(*R*)-Rg_3_ and 20(*S*)/(*R*)-Rh_1_ [30]. The 20(*S*) saponin content was higher than that of 20(*R*) saponin in this process. 

### 2.2. Effects of Single Factors

#### 2.2.1. Effect of Solvent 

**Figure 2:**
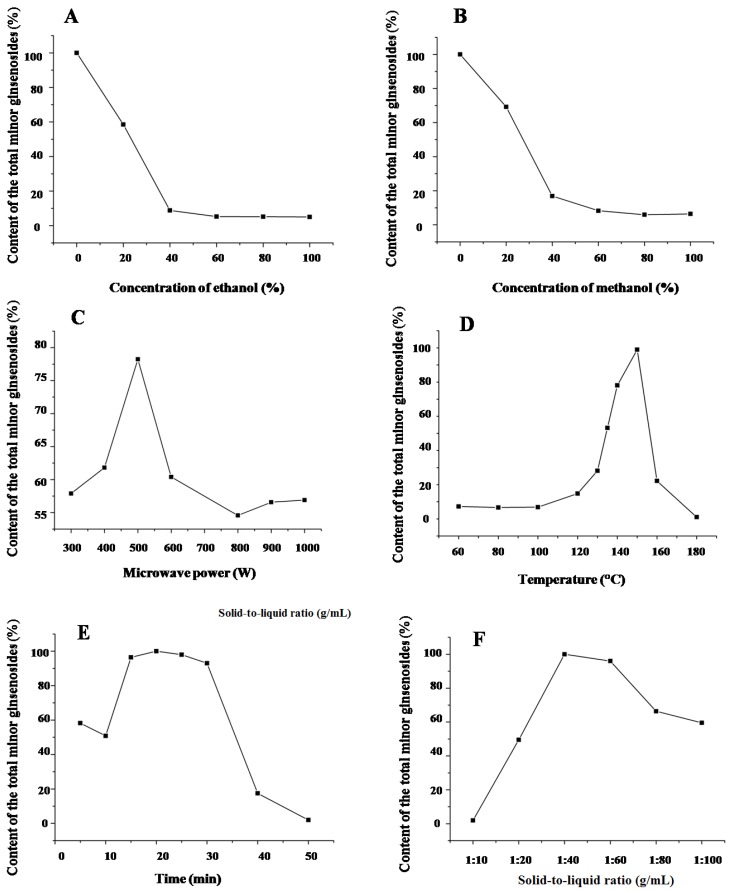
Effect of solvent, microwave power, temperature, time and solid-to-liquid ratio on production of the total minor ginsenosides. (**A**) Ethanol concentration; (**B**) Methanol concentration; (**C**) Microwave power; (**D**) Microwave temperature; (**E**) Microwave time; (**F**) Solid-to-liquid ratio.

In this paper, firstly the solvent used in the microwave processing method was studied. When different concentrations of ethanol or methanol were used, the total yield of the minor ginsenosides (sum of eight saponins 20(*S*)-Rh_1_, 20(*R*)-Rh_1_, Rk_3_, Rh_4_, 20(*S*)-Rg_3_, 20(*R*)-Rg_3_, Rk_1_ and Rg_5_) was different (Figure 2A,B). The results showed that the yield decreased with the increase of ethanol or methanol ratio, when other conditions of microwave power, temperature, time and solid-to-liquid ratio were 500 W, 135 °C, 15 min and 1:60, respectively, so water was thought to be the best solvent in the green microwave extraction. The preparation of the minor ginsenosides without any organic solvent in microwave processing method reduces the environmental burden.

#### 2.2.2. Effect of Microwave Power 

Microwave power was an important factor in any microwave processing method. When the other conditions of temperature, time and solid-to-liquid ratio were 150 °C, 15 min and 1:60, respectively, the microwave power was varied from 300 W to 1000 W (Figure 2C). When the microwave power was lower than 300 W or higher than 600 W, the conversion yield of the total minor ginsenosides was less than 65%. Finally, the optimal microwave power was set at 500 W.

#### 2.2.3. Effect of Temperature 

In order to further study the temperature of microwave processing method, the temperature range was from 60 °C to 180 °C (Figure 2D). The other conditions of microwave power, time and solid-to-liquid ratio were set at 500 W, 15 min and 1:60, respectively. The yield of the total minor ginsenosides was obviously increased when temperature was increased to 150 °C, so it is concluded that temperature had an important influence on the transformation of ginsenosides and 150 °C was the appropriate temperature for the transformation method.

#### 2.2.4. Effect of Microwave Time 

When the microwave power, temperature and solid-to-liquid ratio were 500 W, 150 °C and 1:60, respectively, the microwave time was varied from 5 min to 50 min (Figure 2E). According to the yield of the total minor ginsenosides, a suitable time for this method ranged from 15 min to 35 min. When the microwave exposure time was 15 min, the yield of the total minor ginsenosides was more than 90%. If the conventional steaming method was used, it took at least 4 h to achieve the same yield. The steaming method is usually a highly time and energy consuming process. Compared with the steaming method, the minor ginsenosides were produced by the microwave method with less energy and less waste.

#### 2.2.5. Effect of Solid-to-Liquid Ratio 

While the other optimized conditions were microwave power of 500 W, temperature 150 °C and 20 min time, t solid-to-liquid ratios of 1:10, 1:20, 1:40, 1:60, 1:80, and 1:100 (*w*/*v*) were examined. When the volume of water was 10 mL (ratio 1:40 or 1:60), the total minor ginsenosides had a higher transformation yield (Figure 2F). 

In conclusion, considering a variety of factors, we obtained that optimal conditions for the microwave processing method. Water was selected as the solvent, and other conditions of microwave power, temperature, time and solid-to-liquid ratio were 500 W, 150 °C, 15 min–35 min and 1:40, respectively. Next, based on the single factor analysis for production of the minor ginsenosides by microwave processing, the three most important variables such as microwave power, temperature and time were further investigated by RSM. 

### 2.3. Response Surface Optimization of the Minor Ginsenosides Production

#### 2.3.1. Fitting the Model

Response Surface Methodology (RSM) is widely used for optimization of minor ginsenosides production [31,32] and understanding the relationships among variables. Table 1 shows the different combinations of variables and the nine responses in the Box-Behnken design used. Nine responses in the experimental design, 20(*S*)-Rh_1_ content (*Y*_1_), 20(*R*)-Rh_1_ content (*Y*_2_), Rk_3_ content (*Y*_3_), Rh_4_ content (*Y*_4_), 20(*S*)-Rg_3_ content (*Y*_5_), 20(*R*)-Rg_3_ content (*Y*_6_), Rk_1_ content (*Y*_7_), Rg_5_ content (*Y*_8_) and total minor ginsenosides content (*Y*_9_) were measured. In these treatments, treatment 10 (500 W, 150 °C, 20 min) achieved the highest yield of total minor ginsenosides (95.74%) and treatment 3 (600 W, 160 °C, 20 min) achieved the lowest yield (8.14%). The nine responses in the design were analyzed using analysis of variance (ANOVA). The regression coefficients of second order polynomial models for response variables (*Yi*) are listed in Table 2. For example, among the coefficients for total minor ginsenosides (*Y*_9_), some factors (*β*_12_, *β*_11_, *β*_22_ and *β*_33_) were considered as important factors because their *p*-value was less than 0.05. The following final regression Equation (1) for production of total minor ginsenosides (*Y*_9_) was obtained in terms of coded factors:

*Y* = 92.8 − 2.16*X*_1_ + 5.82*X*_2_ + 2.46*X*_3_ − 9.79*X*_1_*X*_2_ + 3.45*X*_1_*X*_3_ − 0.88*X*_2_*X*_3_ − 26.25*X*_1_^2^ − 46.02*X*_2_^2^ − 17.31*X*_3_^2^ (1)

**Figure 3:**
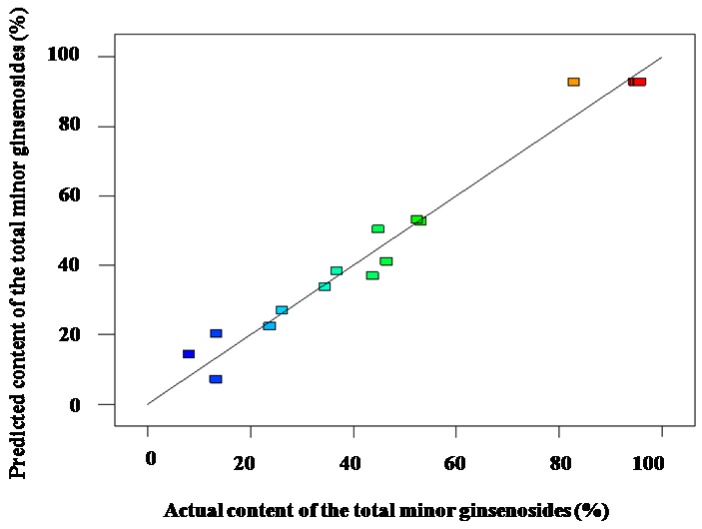
Correlation between actual and predicted values of response (*Y*_9_).

The ANOVA results (Table 3) showed that the total model for production of the minor ginsenosides (*Y*_1_–*Y*_9_) was highly significant, with a *p* value of <0.001, while the lack of fit is not significant relative to the pure error, with a *p* value of >0.05. Hence, this model could be used for predicting all points. Figure 3 further shows that the actual values of total minor ginsenosides (*Y_9_*) were very similar to the predicted values. The fitting degree of the model was checked by regression coefficient (*R*^2^ = 0.9766), and this is in agreement with the adjusted *R*^2^ value of 0.9466. 

According to the regression coefficient results in Table 2, the second-order variables microwave power (*X*_11_) with significance (*p* < 0.05) and temperature (*X*_22_) with high significance (*p* < 0.001) showed a negative effect on yield of the minor ginsenosides (*Y*_1_–*Y*_9_), while the interaction variables *X*_12_ between microwave power and temperature had a significantly negative effect on Rh_4_ yield (*Y*_4_), Rg_5_ yield (*Y*_8_) and yield total minor ginsenosides (*Y*_9_) (*p* < 0.05), *X*_13_ between microwave power and time had a positive effect on 20(*S*)-Rh_1_ yield (*Y*_1_), 20(*R*)-Rh_1_ yield (*Y*_2_), 20(*S*)-Rg_3_ yield (*Y*_5_), 20(*R*)-Rg_3_ yield (*Y*_6_) and Rk_1_ yield (*Y*_7_).

#### 2.3.2. Response Surface Method Analysis 

**Figure 4:**
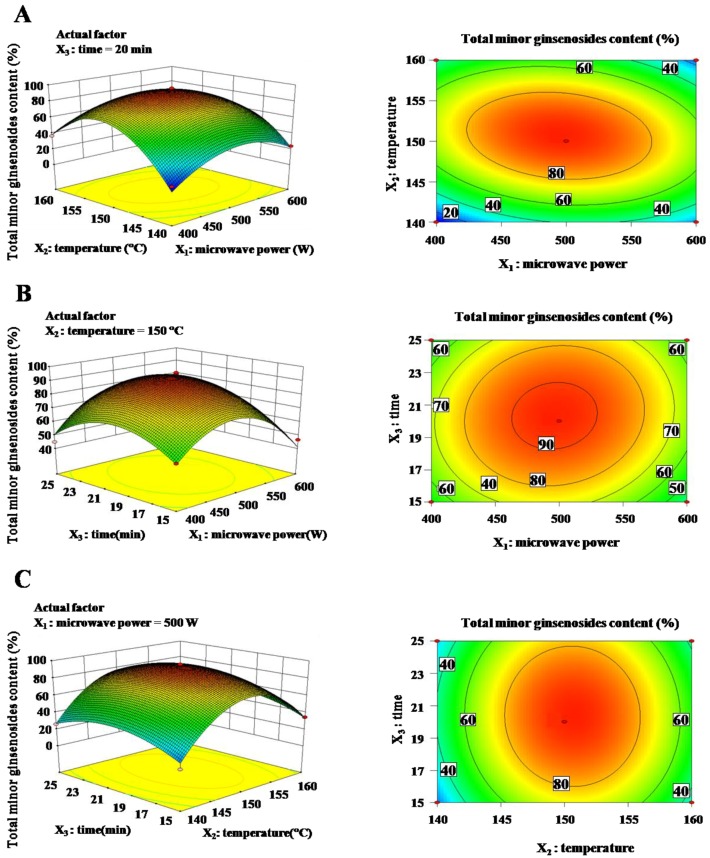
Response surface plots and contour plots for production of the total minor ginsenosides using interaction of variables. (**A**) Effect of microwave power (*X*_1_) and temperature (*X*_2_) on total minor ginsenosides content (*Y*_9_) at time 20 min; (**B**) Effect of microwave power (*X*_1_) and time (*X*_3_) on total minor ginsenosides content (*Y*_9_) at temperature 150 °C; (**C**) Effect of temperature (*X*_2_) and time (*X*_3_) on total minor ginsenosides content (*Y*_9_) at microwave power 500 W.

Response surface and contour plots for production of the total minor ginsenosides based on the interaction between the variables are shown in Figure 4. The relationships between variables and response can be better understood by response surface plots (3D). The content of total minor ginsenosides firstly increased and then decreased with the increase of microwave power, process temperature and time. The shape of contour plots indicated the effect of the interaction between the variables. From Figure 4A, the ellipse-shaped contour plots showed that the interaction of microwave power and processing temperature had a strong influence on the production of the total minor ginsenosides.

#### 2.3.3. Prediction and Verification of the Optimum Conditions

Microwave processing conditions were optimized using response surface methodology (Table 4). For example, the predicted maximum yield of the total minor ginsenosides was 93.13%, when the optimum conditions of microwave power, temperature and time were 495.03 W, 150.68 °C and 20.32 min, respectively. The verification test was carried out three times and compared with the predicted values obtained from the model (Equation (2)). In the optimum conditions, the actual yield of total minor ginsenosides was 94.15% that agree with the predicted values. Therefore, RSM can effectively optimize the production of the minor ginsenosides.

#### 2.3.4. Industrial Feasibility of Production of Minor Ginenosides with the Microwave Processing Method

Microwave technology is widely applied for improving extraction of plant secondary metabolites from leaves, flowers and seeds [33]. The first important contribution of microwave technology from lab to industrialization was the application of the extraction of volatile organic compounds from Boldo leaves [34]. Comparing the microwave method to conventional hydrodistillation, the volatile oil was extracted with less time and energy. In our work, ginsenoside transformation using the microwave method was completed in 20 min, while the conventional steam processing took 4 h. Using this method, the energy efficiency is higher and time consumption is less. The sample thickness is the most important factor in the process of large-scale extraction, because uniform heating is rarely achievable in the microwave method [35]. Under the optimized solid-to-liquid ratio conditions, we found that the crude extract of *P. notoginseng* was completely dissolved in the solvent water before microwave treatment. This result overcame the problem of uneven heating resulting from low penetration depth of microwaves. We have also established HPLC systems suitable for quality control of the transformed products (Table 5), and solved the problem of poor quality control in industrial microwave equipment. Therefore, the scale up of production of minor ginenosides from *Panax notoginseng* in microwave processing method to industrial scale is feasible.

### 2.4. Effect of the Extract of Microwave Processed P. notoginseng on Blood Deficient Mice In Vivo

As previously stated, raw notoginseng has the effect of promoting blood circulation, and steaming notoginseng has the effect of enriching blood [1]. In our work, the effects of raw notoginseng and processed notoginseng prepared by steaming and microwave transformation methods on the peripheral blood index of *N*-acetyl phenylhydrazine (APH) and cyclophosphamide (CTX)-induced blood deficient mice were investigated (Table 6). 

**Figure 5:**
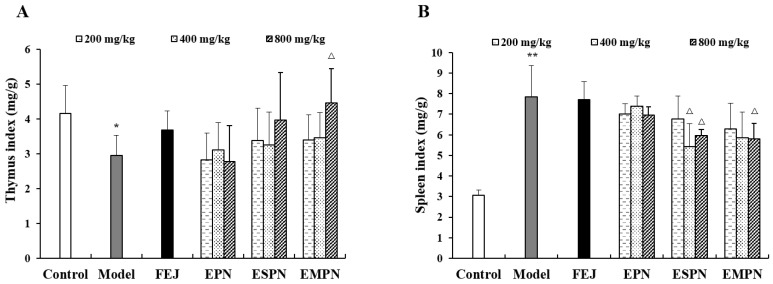
Effect of raw notoginseng and processed notoginseng on thymus index (**A**) and spleen index (**B**) of blood deficiency model mice. FEJ, Fufang E’jiao Jiang; EPN, the extract of *P. notoginseng*; ESPN, the extract of steamed *P. notoginseng*; EMPN, the extract of microwave processed *P. notoginseng.* Data are expressed as mean ± *s* (*n* = 10 in group). Compared with the negative control group: * *p* < 0.05, ** *p* < 0.01; compared with the model group: ^Δ^ *p* < 0.05.

The concentration of white blood cells (WBC), red blood cells (RBC) and hemoglobin (HGB) and hematocrit (HCT) are usually used in the clinical diagnosis of anemia [36]. Compared with normal control group, the content of WBC (*p <* 0.001), RBC (*p <* 0.01) and HGB (*p <* 0.001) and hematocrit (HCT) (*p <* 0.05) in model group decreased significantly, which suggested that establishment of this blood deficiency model was effect. WBC and HGB in extract of steamed *P. notoginseng* (ESPN) and extract of microwave processed *P. notoginseng* (EMPN) groups showed a significant increase trend compared with model group, suggesting processed notoginseng could improve the symptoms of blood deficiency. And in the high dose group (800 mg/kg), the results showed the best effect, which were similar to the positive sample of Fufang E’jiao Jiang (FEJ). Fufang E’jiao Jiang in the Chinese Pharmacopoeia (2015 edition) is used to treat qi-blood deficiency, dizziness, loss of appetite, leucopenia and anemia. However, raw notoginseng (EPN) had no effect on reduced WBC, RBC, HGB, and HCT. The combination of APH and CTX reduced the number of RBC in the blood, and damaged the immune organs of mice [37]. Furthermore, we studied the effect of raw notoginseng (EPN) and processed notoginseng (ESPN and EMPN) on immune organs of blood deficient mice (Figure 5). Compared with the normal control group, the thymus index in the model group decreased significantly (*p <* 0.05), and the spleen index increased significantly (*p <* 0.01). The results showed that there was no effect on the thymus index and spleen index in the EPN group. But ESPN at the dose of 400 mg/kg and 800 mg/kg reduced the increasing spleen index (*p <* 0.05). EMPN at the dose of 800 mg/kg significantly improved thymus index (*p <* 0.05) and decreased spleen index (*p <* 0.05) of blood deficiency mice. EMPN had protective effect on the immune organs of mice.

### 2.5. Effect of the Extract of Microwave Processed P. notoginseng on Hemostatic Activity In Vitro

**Figure 6:**
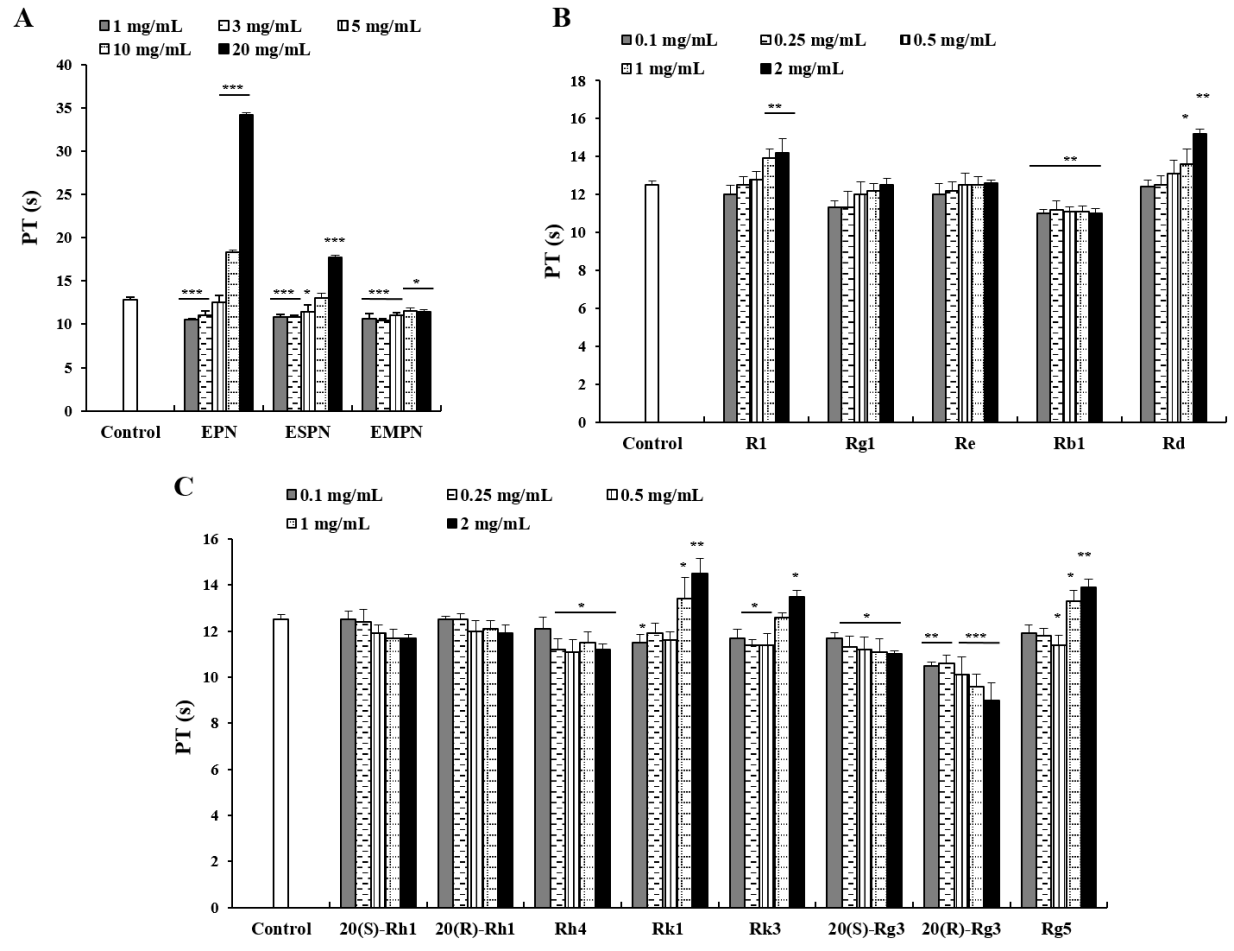
Hemostatic activities of raw notoginseng and processed notoginseng. (**A**) PT assays of the extract of *P. notoginseng* (EPN), the extract of steamed *P. notoginseng* (ESPN) and the extract of microwave processed *P. notoginseng* (EMPN); (**B**) PT assays of ginsenosides Rg_1_, Re, Rb_1_, Rd and notoginsenoside R_1_; (**C**) PT assays of ginsenosides 20(*S*)-Rh_1_, 20(*R*)-Rh_1_, Rh_4_, Rk_1_, Rk_3_, 20(*S*)-Rg_3_, 20(*R*)-Rg_3_ and Rg_5_. Data are expressed as mean ± *s* (*n* = 3). Compared with the negative control group: * *p* < 0.05, ** *p* < 0.01, *** *p* < 0.001.

PT value reflects the activity of extrinsic coagulation system [38]. The hemostatic activity of EPN, ESPN, EMPN and their main saponins is shown in Figure 6. Compared with the negative control, EPN and ESPN had a two-way regulatory effect of hemostasis and activating blood circulation. EPN and ESPN had hemostatic effect significantly (*p* < 0.001) at the low concentration of 1 mg/mL and 3 mg/mL. EPN and ESPN has significant anticoagulant effect (*p* < 0.001), when using the high concentration of 20 mg/mL (Figure 6A). However, EMPN showed the hemostatic effect at a different concentration. The main components of raw notoginseng (EPN) were ginsenosides Rg_1_, Re, Rb_1_, Rd and notoginsenoside R_1_. PT testing results showed that ginsenosides R_1_ and Rd significantly prolonged the clotting time (*p* < 0.01), and ginsenoside Rb_1_ shortened the clotting time at the concentration of 2 mg/L (Figure 6B). It is reported that Rb_1_ has the effect of antiplatelet aggregation [39]. No statistically significant effect on PT value was observed for the other compounds of Rg_1_ and Re. The main components in ESPN or EMPN are ginsenosides 20(*S*)-Rh_1_, 20(*R*)-Rh_1_, Rk_3_, Rh_4_, 20(*S*)-Rg_3_, 20(*R*)-Rg_3_, Rk_1_ and Rg_5_. The PT values of these eight compounds are shown in Figure 6C. When the concentration of ginsenosides was 2 mg/mL, the PT time of Rk_1_ and Rg_5_ was prolonged (*p* < 0.01), and the PT time of Rh_4_ (*p* < 0.05), 20(*S*)-Rg_3_ (*p* < 0.05), 20(*R*)-Rg_3_ (*p* < 0.001) was shortened. The results showed that the hemostatic activity of raw notoginseng (EPN) and processed notoginseng (ESPN and EMPN) were significantly different, which was related to their different chemical composition. The hemostatic effect of EMPN is mainly caused by its components of Rh_4_, 20(*S*)-Rg_3_, 20(*R*)-Rg_3_.

## 3. Materials and Methods

### 3.1. Chemicals and Materials

Standard ginsenosides Re (lot number: 20141015), Rg_1_ (lot number: 20140809), Rb_1_ (lot number: 20140828), Rd (lot number: 20141105), 20(*S*)-Rg_3_ (lot number: 20141109), 20(*R*)-Rg_3_ (lot number: 20141205) and notoginsenoside R_1_ (lot number: 20140925) were purchased from Jinsui Bio-technology Co. Ltd. (Shanghai, China). Standard ginsenoside 20(*S*)-Rh_1_ (lot number: MUST-13052205) was purchased from Must Bio-technology Co. Ltd. (Chengdu, China). Standard ginsenoside 20(*R*)-Rh_1_ (lot number: JBZ-1065) was purchased from Nanjing Jin Yibai Biological Technology Co. Ltd. (Nanjing, China). Standard ginsenosides Rk_3_ (lot number: GR-133-140724) and Rh_4_ (lot number: GR-133-130721) were purchased from Guangrun Bio-technology Co. Ltd. (Nanjing, China). Standard ginsenosides Rk_1_ (lot number: P20N6F6254), Rg_5_ (lot number: P20N6F6253), cyclophosphamide (lot number: SJ0121RA14) and *N*-acetylphenylhydrazine (lot number: AA14446) were purchased from Yuanye Bio-technology Co. Ltd. (Shanghai, China). Fufang E’jiao Jiang (lot number: 160519) were purchased from Shandong Fujiao Group Co., Ltd. (Jinan, China). The PT kit was purchased from Wuhan Zhongtai Biotech Co. Ltd. (Wuhan, China). Acetonitrile (HPLC grade) was purchased from Sigma Aldrich (Saint Louis, MO, USA) and other solvents (A.R.) were purchased from Fengchuan Chemical Reagent Technology Co. Ltd. (Tianjin, China).

### 3.2. Preparation of Processed notoginseng Using the Microwave Processing Method

The dried roots of *P. notoginseng* were purchased from the Wenshan market in Yunnan Province, China. The roots were ground to pass through a 40-mesh sieve and extracted ultrasonically with 70% aqueous MeOH, three times for 45 min. After filtration, filtrate was evaporated to give the extract of *P. notoginseng* (32% of dried weight). The extract (250 mg) was added to the solvent (10 mL) in a microwave extraction system (model no.: MDS-6G) manufactured by Sineo Microwave Chemical Technology Co. Ltd. (Shanghai, China). This microwave instrument has a high precision platinum electric resistance sensor, which controls the temperature and displays temperature curve in real time. The temperature range is 0–300 °C. The solution was irradiated by microwave in sealed containers for the different microwave power, temperature and time. The solution was centrifuged at 500× *g* for 5 min and the supernatant was added the solvent to 10 mL. This sample solution was prepared for HPLC analysis.

### 3.3. HPLC Analysis of Ginenosides

By comparing with the retention time of standard ginsenosides in HPLC, eight new transformed ginsenosides of *P. notoginseng* were identified as 20(*S*)-Rh_1_, 20(*R*)-Rh_1_, Rh_4_, Rk_1_, Rk_3_, 20(*S*)-Rg_3_, 20(*R*)-Rg_3_ and Rg_5_ (Figure 1). Quantitative analysis of the eight minor ginsenosides processed by microwave method was determined by HPLC. HPLC was performed by a Shimadzu Analytical Instrument (Shimadzu, Kyoto, Japan), equipped with a Shimadzu, DGU-20A3R(C) solvent degasser, Shimadzu, LC-20AB binary pump, a Shimadzu SIL-20A auto sampler and a Shimadzu SPD-20A UV detector. The detection wavelength was 203 nm, and the column temperature was at 35 °C. The column was the Vision HT C18 (250 mm × 4.6 mm, 5 μm). The mobile phase consisted of distilled water (solvent A) and acetonitrile (solvent B) at the flow rate of 1.0 mL/min. The solvent B ratios were as follows: 20–20%, 20–46%, 46–55%, 55–55% with retention times of 0 min–20 min, 20 min–45 min, 45 min–50 min, 50 min–60 min, 60 min–65min respectively. The method was validated for the determination of eight minor ginsenosides (Table 5). The calibration curves for 20(*S*)-Rh_1_, 20(*R*)-Rh_1_, Rh_4_, Rk_1_, Rk_3_, 20(*S*)-Rg_3_, 20(*R*)-Rg_3_ and Rg_5_ showed good linearity (*R*^2^ > 0.9990) in the concentration ranges.

### 3.4. Experimental Design

#### 3.4.1. Single-Factor Experiments

Next microwave processed products were obtained in different conditions. The single factor conditions for the minor ginsenosides production were studied. Solvents with different concentrations of ethanol or methanol (from 0% to 100%) were used. Considering the influence of the ratio of material to volume, the ratios of 1:10, 1:20, 1:40, 1:60, 1:80, and 1:100 (*w*/*v*) were selected. When the solvent is water and material ratio is 1:60, the temperature ranged from 60 °C to 180 °C, microwave power ranged from 300 W to 1000 W, and time ranged from 5 min to 50 min. The obtained samples were filtered through a 0.45 μm filter membrane for HPLC analysis.

#### 3.4.2. Response Surface Methodology

According to the results of single factor analysis, the three most important independent variables, *X*_1_ (microwave power), *X*_2_ (temperature) and *X*_3_ (time) were chosen for further evaluation in response surface methodology (RSM). Table 1 shows different combinations of the independent factors (*Xi*) and actual experimental responses (*Yi*) using Box-Behnken Design. The responses included 20(*S*)-Rh_1_ content (*Y*_1_), 20(*R*)-Rh_1_ content (*Y*_2_), Rk_3_ content (*Y*_3_), Rh_4_ content (*Y*_4_), 20(*S*)-Rg_3_ content (*Y*_5_), 20(*R*)-Rg_3_ content (*Y*_6_), Rk_1_ content (*Y*_7_), Rg_5_ content (*Y*_8_) and total minor ginsenosides content (*Y*_9_). The predicted responses were calculated by the second degree polynomial Equation (2):

*Y* = *β*_0_ + *β*_1_*X*_1_ + *β*_2_*X*_2_ + *β*_3_*X*_3_ + *β*_11_*X*_1_^2^ + *β*_22_*X*_2_^2^ + *β*_33_*X*_3_^2^ + *β*_12_*X*_1_*X*_2_ + *β*_13_*X*_1_*X*_3_ +*β*_23_*X*_2_*X*_3_ + *ε* (2)

The coefficients of the polynomial were intercept (*β*_0_), linear coefficients (*β*_1_, *β*_2_, *β*_3_), squared coefficients (*β*_11_, *β*_22_, *β*_33_) and interaction coefficients (*β*_12_, *β*_13_, *β*_23_). Graphical analysis of data was completed by Design Expert trial 8.0.5 (Stat-Ease, Minneapolis, MN, USA). Response surface quadratic model was analyzed by variance analysis (ANOVA). The significance of model was checked by *R*^2^, adjusted *R*^2^ and goodness of fit. *P* values of less than 0.05 were considered significant.

### 3.5. Pharmacological Assays

#### 3.5.1. Animals

Kunming mice (18–22 g) of both sexes were supplied by Changsha Tianqin Biotechnology Co., (Changsh, China). The approval number is SCXK 2014-0011. The mice were kept at 25 ± 2 °C and a 12 h dark/light cycle condition. All experimental protocols were approved by the Animal Ethical Committee of Laboratory Animals of Kunming University of Science and Technology. 

#### 3.5.2. Extract Preparation

The roots of *P. notoginseng* (500 g) were powdered and ultrasonically extracted with 70% aqueous MeOH, three times for 45 min. After filtration, the solvent was evaporated to give the extract of *P. notoginseng* (EPN, 162.3 g) with a yield of 32.5%.

Powdered roots of *P. notoginseng* (500 g) were steamed at 120 °C for 4 h [23] and ultrasonically extracted with 70% aqueous MeOH, three times for 45 min. After filtration and concentration, the extract of steamed *P.* notoginseng (ESPN, 125.4 g) was obtained with a yield of 25.1%. 

The methonal extract of *P. notoginseng* was processed using optimal microwave conversion conditions. The EPN (50 g) was irradiated with microwave at a power of 500 W and a temperature of 150°C for 20 min to give the extract of microwave processed *P.* notoginseng (EMPN, 47.1 g) with a yield of 94.2%.

#### 3.5.3. *N*-Acetyl Phenylhydrazine (APH) and Cyclophosphamide (CTX) Induced Blood Deficiency in Mice

The establishment of the blood deficiency model was used according to the method described by Li et al. [36]. Mice (120) were randomly divided into 12 groups with 10 mice in each group, i.e., normal control group (Control); blood deficiency model group (Model); positive group of Fufang E’jiao Jiang (FEJ, 10 mg/kg); low dose group of EPN (200 mg/kg), middle dose group of EPN (400 mg/kg), high dose group of EPN (800 mg/kg); low dose group of ESPN (200 mg/kg), middle dose group of ESPN (400 mg/kg), high dose group of ESPN (800 mg/kg); low dose group of EMPN (200 mg/kg), middle dose group of EMPN (400 mg/kg), high dose group of EMPN (800 mg/kg). The FEJ, EPN, ESPN and EMPN were intragastrically administered in each group, once a day for two weeks. The normal control group and model group were given an equal volume of normal saline. Except for the normal control group, mice in each group were hypodermically injected with 2% APH saline solution (20 mg/kg) on the 1st day, and intraperitoneally injected with CTX saline solution (70 mg/kg) on the 2st day. After 7 days of continuous injection, the blood deficiency model was established. Orbit blood samples of mice was collected on day 7 for detecting the white blood cell (WBC), red blood cell (RBC), hemoglobin (HGB), and hematocrit (HCT). After collection of blood, the mice were weighed and killed by cervical dislocation. The thymus and spleen were removed and weighed. The spleen or thymus index was calculated according to the following formula:

Spleen or thymus index (mg/g) = (spleen weight or thymus weight/body weight)

#### 3.5.4. Blood Plasma Clotting Analysis

Prothrombin time (PT) was measured using a coagulation analyzer (XN06-IV, Aierfu, Wuhan, China). The plasma was obtained from mice whole blood added with 0.109 mol/L sodium citrate (9:1 ratio of blood to citrate) by centrifugation at 1000× *g* for 15 min. The mixtures of the plasma (50 μL) with EPN, ESPN, EMPN or their compounds (50 μL) were incubated at 37 °C for 3 min. The samples of EPN, ESPN and EMPN were diluted with purified water to give the five concentrations: 20, 10, 5, 3, 1 mg/mL. The ginsenosides Rg_1_, Rb_1_, Re, Rd, 20(*S*)-Rh_1_, 20(*R*)-Rh_1_, Rh_4_, Rk_1_, Rk_3_, 20(*S*)-Rg_3_, 20(*R*)-Rg_3_ and Rg_5_ and notoginsenoside R_1_ were diluted to other five concentrations: 2, 1, 0.5, 0.25, 0.1 mg/mL. The PT assay reagent (100 μL) was added to the mixed samples and the clotting time was recorded. Purified water was used as negative control.

#### 3.5.5. Statistical Analysis

Statistical analyses were performed with SPSS 18.0 software (SPSS Inc., Chicago, IL, USA), and the data were expressed as mean ± s. Single factor analysis of variance was used to compare between groups. The values of *p* < 0.05 were considered to be statistically significant, and *p* < 0.01 and *p* < 0.001 being very significant.

## 4. Conclusions

In this study, the generated minor ginsenosides from *P. notoginseng* can be used to illustrate the transformation of ginsenosides during microwave processing. Eight minor ginsenosides from *P. notoginseng* were identified as ginsenosides 20(*S*)-Rh_1_, 20(*R*)-Rh_1_, Rh_4_, Rk_1_, Rk_3_, 20(*S*)-Rg_3_, 20(*R*)-Rg_3_ and Rg_5_. When the microwave power and temperature were increased, the glycosyl residues of major saponins were easily decreased. 

Eight minor ginsenosides was successfully prepared from *P. notogingseng* by microwave processing and the transformation conditions were optimized by RSM. The experimental results showed that the yield of the total minor gisenosides was 93.13% when the optimum conditions of microwave power, temperature and time were 495.03 W, 150.68 °C and 20.32 min, respectively. Under the optimum conditions, the actual yield of total minor ginsenosides was 94.15% that agreed with the predicted values. The RSM in this study was effective to optimize the production of the minor ginsenosides. 

The pharmacological results of the extract of microwave processed *P. notoginseng* (EMPN) in vivo showed that EMPN had the effect of enriching blood, similar to the extract of steamed *P. notoginseng* (ESPN) in APH and CTX induced blood deficient mice. WBC and HGB in EMPN group showed a significant increase trend compared with the model group. EMPN had a protective effect on the immune organs of mice. Meanwhile, the in vitro hemostatic activity of EMPN showed that it had significantly shortened the clotting time on PT testing. The hemostatic effect of EMPN is mainly caused by its components of Rh_4_, 20(*S*)-Rg_3_, 20(*R*)-Rg_3_. 

Therefore, this microwave transformed method was simple and suitable to mass-produce the minor ginsenosides on an industrial scale and conducive to studying their blood-enriching and hemostatic activity.

## Figures and Tables

**Table 1:** Experimental design factors and response values in Box-Behnken Design.

No.	Independent Variables			Response Variables								
	Microwave Power (*X*_1_, W)	Temperature (*X*_2_, °C)	Time (*X*_3_, min)	20(*S*)-Rh_1_ (*Y*_1_, %)	20(*R*)-Rh_1_ (*Y*_2_, %)	Rk_3_ (*Y*_3_, %)	Rh_4_ (*Y*_4_, %)	20(*S*)-Rg_3_ (*Y*_5_, %)	20(*R*)-Rg_3_ (*Y*_6_, %)	Rk_1_ (*Y*_7_, %)	Rg_5_ (*Y*_8_, %)	Total Minor Ginsenosides (*Y*_9_^a^, %)
1	600 (1)	150 (0)	25 (1)	8.49	4.23	3.89	13.31	2.87	1.17	7.63	10.85	52.45
2	500 (0)	160 (1)	25 (1)	7.63	2.04	2.74	12.59	1.92	0.90	6.29	9.66	43.76
3	600 (1)	160 (1)	20 (0)	0.86	1.29	0.87	1.07	1.35	0.90	0.77	1.04	8.14
4	400 (−1)	140 (−1)	20 (0)	1.51	2.27	1.03	3.13	2.45	0.79	0.90	1.26	13.34
5	400 (−1)	160 (1)	20 (0)	4.24	1.72	6.03	11.86	1.75	0.79	3.02	7.41	36.83
6	500 (0)	160 (1)	15 (−1)	2.95	2.23	7.42	10.93	1.93	1.09	1.03	6.97	34.53
7	400 (−1)	150 (0)	25 (1)	5.97	0.62	6.14	14.42	0.96	0.29	4.68	11.80	44.88
8	600 (1)	140 (−1)	20 (0)	5.41	1.54	2.84	5.85	1.24	0.40	2.69	3.82	23.80
9	500 (0)	150 (0)	20 (0)	14.25	7.00	14.76	24.22	6.51	3.65	12.20	13.01	95.61
10	500 (0)	150 (0)	20 (0)	14.17	7.77	13.52	22.05	5.80	4.03	12.53	15.88	95.74
11	500 (0)	150 (0)	20 (0)	15.76	7.17	15.80	20.73	6.12	4.28	9.85	14.97	94.67
12	500 (0)	150 (0)	20 (0)	14.85	8.55	11.52	21.62	4.46	4.61	13.17	16.28	95.05
13	600 (1)	150 (0)	15 (−1)	2.30	1.31	9.54	17.76	1.03	0.84	1.46	12.27	46.51
14	400 (−1)	150 (0)	15 (−1)	7.69	3.75	7.26	12.55	2.81	3.79	6.44	8.82	53.12
15	500 (0)	140 (−1)	25 (1)	1.63	0.83	6.07	8.91	1.05	1.24	0.73	5.69	26.15
16	500 (0)	140 (−1)	15 (−1)	1.13	1.02	0.84	5.45	0.93	0.22	0.52	3.28	13.41
17	500 (0)	150 (0)	20 (0)	13.06	5.21	10.17	17.74	4.92	4.92	11.48	15.44	82.93

^a^ Total minor ginsenosides content (*Y*_9_), Sum of eight saponins 20(*S*)-Rh_1_, 20(*R*)-Rh_1_, Rk_3_, Rh_4_,20(*S*)-Rg_3_, 20(*R*)-Rg_3_, Rk_1_ and Rg_5_.

**Table 2:** Regression coefficients of second order polynomial for response variables.

Variable	Coefficient (*β*)									*p*-Value ^a^								
	*Y*_1_	*Y*_2_	*Y*_3_	*Y*_4_	*Y*_5_	*Y*_6_	*Y*_7_	*Y*_8_	*Y*_9_	*Y*_1_	*Y*_2_	*Y*_3_	*Y*_4_	*Y*_5_	*Y*_6_	*Y*_7_	*Y*_8_	*Y*_9_
Intercept	14.42	7.14	13.15	21.27	5.56	4.29	11.85	15.12	92.8	0.0003	0.0027	0.004	0.0025	0.0023	0.0028	0.0002	0.0003	<0.0001
*X*_1_	−0.29	0.0018	−0.41	−0.50	−0.19	−0.29	−0.31	−0.16	−2.16	0.6100	0.9963	0.5849	0.6218	0.5094	0.2698	0.5068	0.7649	0.4236
*X*_2_	0.75	0.20	0.78	1.64	0.16	0.13	0.78	1.38	5.82	0.2156	0.6135	0.3150	0.1316	0.5736	0.6211	0.1233	0.0338	0.0558
*X*_3_	1.21	−0.075	−0.78	0.32	0.013	−0.29	1.23	0.83	2.46	0.0645	0.8490	0.3187	0.7503	0.9628	0.2733	0.0283	0.1565	0.3654
*X*_12_	−1.82	0.075	−1.74	−3.38	0.20	0.12	−1.01	−2.23	−9.79	0.0520	0.8924	0.1327	0.0417	0.6120	0.7345	0.1546	0.0194	0.0296
*X*_13_	1.98	1.51	−1.13	−1.58	0.92	0.96	1.98	−1.10	3.54	0.0386	0.0261	0.3052	0.2831	0.0457	0.0279	0.0166	0.1810	0.3567
*X*_23_	1.04	0.0012	−2.48	−0.45	−0.032	−0.30	1.26	0.071	−0.88	0.2223	0.9982	0.0463	0.7506	0.9362	0.4108	0.0860	0.9259	0.8141
*X*_11_	−4.32	−2.24	−4.01	−5.38	−1.70	−1.46	−3.55	−3.60	−26.25	0.0007	0.0037	0.0051	0.0048	0.0025	0.0036	0.0007	0.0016	0.0001
*X*_22_	−7.09	−3.19	−6.45	−10.42	−2.16	−2.12	−6.45	−8.13	−46.03	<0.0001	0.0005	0.0003	0.0001	0.0006	0.0004	<0.0001	<0.0001	<0.0001
*X*_33_	−3.99	−2.42	−2.44	−1.38	−1.94	−1.31	−3.25	−0.58	−17.31	0.0012	0.0024	0.0448	0.3303	0.0012	0.0060	0.0012	0.4467	0.0017

^a^*p* < 0.01, highly significant; 0.01 < *p* < 0.05 significant; *p* > 0.05, not significant.

**Table 3:** ANOVA for response surface quadratic model analysis.

Variable	Degree of Freedom	Sum of Squares								
		*Y*_1_	*Y*_2_	*Y*_3_	*Y*_4_	*Y*_5_	*Y*_6_	*Y*_7_	*Y*_8_	*Y*_9_
Model ^a^	9	445.35	108.39	346.55	707.60	57.37	44.58	344.40	399.30	15,109.32
Residual	7	17.00	8.10	29.39	51.59	4.04	3.38	11.22	15.35	361.53
Lack of fit ^b^	3	13.09	1.97	8.12	29.36	1.14	2.40	4.77	8.85	239.01
Pure error	4	3.91	6.13	21.27	22.23	2.90	0.97	6.45	6.49	122.52
Correct total	16	462.35	116.49	375.94	759.19	61.40	47.95	355.62	414.65	15,470.85
*R*-Squared		0.9632	0.9305	0.9218	0.9320	0.9342	0.9296	0.9684	0.9630	0.9766
Adj *R*-Squared		0.9160	0.8411	0.8213	0.8447	0.8497	0.8391	0.9279	0.9154	0.9466

^a^ Model for *Y*_1_–*Y*_9_ is highly significant (*p* < 0.001); ^b^ Lack of fit for *Y*_1_–*Y*_9_ is not significant (*p* > 0.05).

**Table 4:** Optimum conditions for maximum 20(*S*)-Rh_1_ content, 20(*R*)-Rh_1_ content, Rk_3_ content, Rh_4_ content, 20(*S*)-Rg_3_ content, 20(*R*)-Rg_3_ content, Rk_1_ content, Rg_5_ content and total minor ginsenosides content.

Response Variables (*Y_i_*)	Microwave Power (*X*_1_, W)	Temperature (*X*_2_, °C)	Time (*X*_3_, min)	Content (%)
20(*S*)-Rh_1_ (*Y*_1_)	498.80	150.66	20.79	14.54
20(*R*)-Rh_1_ (*Y*_2_)	499.52	150.31	19.91	7.14
Rk_3_ (*Y*_3_)	495.36	151.07	18.98	13.28
Rh_4_ (*Y*_4_)	490.33	150.91	20.78	21.39
20(*S*)-Rg_3_ (*Y*_5_)	494.40	150.34	19.95	5.57
20(*R*)-Rg_3_ (*Y*_6_)	484.35	150.38	19.13	4.35
Rk_1_ (*Y*_7_)	500.13	150.81	21.03	12.00
Rg_5_ (*Y*_8_)	480.22	151.16	24.55	15.59
Total minor ginsenosides (*Y*_9_)	495.03	150.68	20.32	93.13

**Table 5:** Method validation.

Compounds	Calibration Curve	*R* ^2^	Linear Range (mg/mL)	Precision (RSD, %)	Stability (RSD, %)	Repeatability (RSD, %)	Recovery (mean ± *s*, %)
20(*S*)-ginsenoside Rh_1_	y = 1,000,000x + 52,373	0.9998	0.02–3.20	0.89	3.65	3.58	102.16 ± 2.46
20(*R*)-ginsenoside Rh_1_	y = 2,000,000x + 154,738	0.9994	0.02–3.20	0.79	2.58	3.26	101.58 ± 3.11
ginsenoside Rk_3_	y = 1,000,000x + 22,910	0.9999	0.08–6.40	0.36	2.45	3.31	105.25 ± 3.24
ginsenoside Rh_4_	y = 826,150x − 23,734	0.9996	0.16–9.60	0.45	2.69	3.24	101.78 ± 2.58
20(*S*)-ginsenoside Rg_3_	y = 1,000,000x + 9671.9	0.9999	0.01–3.20	0.48	2.16	3.83	108.48 ± 3.64
20(*R*)-ginsenoside Rg_3_	y = 2,000,000x + 65,498	0.9996	0.005–1.60	0.46	2.28	3.34	104.15 ± 2.64
ginsenoside Rk_1_	y = 1,000,000x + 14881	0.9997	0.08–6.40	0.52	3.97	3.86	103.78 ± 2.89
ginsenoside Rg_5_	y = 301,295x − 8602.8	0.9997	0.08–6.40	0.85	3.41	4.46	105.89 ± 3.66

**Table 6:** Effect of raw notoginseng and processed notoginseng on peripheral blood index of blood deficiency mice (*x* ± *s*, *n* = 10).

Group	Dose (mg/kg)	WBC (10^9^/L)	RBC (10^12^/L)	HGB (g/L)	HCT (%)
Control		7.62 ± 0.66	7.11 ± 0.68	146.8 ± 4.98	33.77 ± 2.36
Model		4.38 ± 0.66 ^△△△^	5.77 ± 0.58 ^△△^	96.2 ± 4.11 ^△△△^	30.4 ± 1.37 ^△^
FEJ	10	5.45 ± 0.42 **	6.81 ± 0.63 *	128.67 ± 8.35 ***	31.07 ± 1.56
EPN	200	4.2 ± 0.18	4.54 ± 1.18	103.18 ± 8.18	30.33 ± 3.78
	400	4.12 ± 0.08	4.76 ± 0.85	107.52 ± 6.96	30.92 ± 1.31
	800	4.07 ± 0.42	5.03 ± 1.61	111.47 ± 14.87	31.63 ± 1.11
ESPN	200	4.35 ± 0.34	4.97 ± 0.69	102.47 ± 18.16	30.12 ± 2.12
	400	5.38 ± 0.26 **	5.93 ± 0.66	124.08 ± 10.7 ***	30.58 ± 2.62
	800	7.10 ± 0.97 ***	6.63 ± 0.83	128.86 ± 16.01 ***	32.3 ± 3.14
EMPN	200	4.16 ± 0.09	4.65 ± 0.43	102.94 ± 6.66	30.74 ± 0.82
	400	5.65 ± 0.23 **	5.24 ± 0.76	105.68 ± 14.11	30.27 ± 1.48
	800	5.55 ± 0.39 **	5.59 ± 0.35	115.63 ± 6.52 ***	32.22 ± 2.67

Control group, *N*-acetyl phenylhydrazine (APH) and cyclophosphamide (CTX) were not induced. Model group, APH and CTX were induced. FEJ positive group, FEJ was administered to APH and CTX induced mice in a dose of 10 mg/kg. EPN group, EPN (200, 400 and 800 mg/kg of body weight) was respectively administered to APH and CTX induced mice. ESPN group, ESPN (200, 400 and 800 mg/kg of body weight) was respectively administered to APH and CTX induced mice. EMPN group, EMPN (200, 400 and 800 mg/kg of body weight) was respectively administered to APH and CTX induced mice. FEJ, Fufang E’jiao Jiang; EPN, extract of *P. notoginseng*; ESPN, extract of steamed *P. notoginseng*; EMPN, extract of microwave processed *P. notoginseng*. WBC, white blood cell; RBC, red blood cell; HGB, hemoglobin; HCT, hematocrit. Compared with normal control group: ^△^ *p <* 0.05, ^△△^ *p <* 0.01, ^△△△^ *p <* 0.001; compared with blood deficiency model group: * *p<* 0.05, ** *p <* 0.01, *** *p <* 0.001.
